# Distinct gene expression signatures induced by viral transactivators of different HTLV-1 subgroups that confer a different risk of HAM/TSP

**DOI:** 10.1186/s12977-018-0454-x

**Published:** 2018-11-06

**Authors:** Tadasuke Naito, Jun-ichirou Yasunaga, Yuichi Mitobe, Kazumasa Shirai, Hiroe Sejima, Hiroshi Ushirogawa, Yuetsu Tanaka, Tatsufumi Nakamura, Kousuke Hanada, Masahiro Fujii, Masao Matsuoka, Mineki Saito

**Affiliations:** 1 Department of Microbiology, Kawasaki Medical School, 577 Matsushima, Kurashiki, Okayama, 701-0192 Japan; 2 Laboratory of Virus Control, Institute for Frontier Life and Medical Sciences, Kyoto University, Kyoto, Japan; 3 Department of Bioscience and Bioinformatics, Kyushu Institute of Technology, Fukuoka, Japan; 4 Department of Immunology, Graduate School of Medicine, University of the Ryukyus, Okinawa, Japan; 5 Department of Social Work, Faculty of Human and Social Studies, Nagasaki International University, 2825-7 Huis Ten Bosch Machi, Sasebo, Nagasaki, 859-3298 Japan; 6 Division of Virology, Niigata University Graduate School of Medical and Dental Sciences, Niigata, Japan; 7 Department of Hematology, Rheumatology and Infectious Disease, Faculty of Life Sciences, Kumamoto University, Kumamoto, Japan; 8 Present Address: Division of Gene Regulation and Signal Transduction, Research Center for Genomic Medicine, Saitama Medical University, Saitama, Japan

**Keywords:** HTLV-1, Virus subgroup, HAM/TSP, Tax, HBZ, Microarray

## Abstract

**Background:**

Among human T cell leukemia virus type 1 (HTLV-1)-infected individuals, there is an association between HTLV-1 *tax* subgroups (subgroup-A or subgroup-B) and the risk of HAM/TSP in the Japanese population. To investigate the role of HTLV-1 subgroups in viral pathogenesis, we studied the functional difference in the subgroup-specific viral transcriptional regulators Tax and HBZ using microarray analysis, reporter gene assays, and evaluation of viral-host protein–protein interaction.

**Results:**

(1) Transcriptional changes in Jurkat Tet-On human T-cells that express each subgroup of Tax or HBZ protein under the control of an inducible promoter revealed different target gene profiles; (2) the number of differentially regulated genes induced by HBZ was 2–3 times higher than that induced by Tax; (3) Tax and HBZ induced the expression of different classes of non-coding RNAs (ncRNAs); (4) the chemokine CXCL10, which has been proposed as a prognostic biomarker for HAM/TSP, was more efficiently induced by subgroup-A Tax (Tax-A) than subgroup-B Tax (Tax-B), in vitro as well as in unmanipulated (ex vivo) PBMCs obtained from HAM/TSP patients; (5) reporter gene assays indicated that although transient Tax expression in an HTLV-1-negative human T-cell line activated the CXCL10 gene promoter through the NF-κB pathway, there was no difference in the ability of each subgroup of Tax to activate the CXCL10 promoter; however, (6) chromatin immunoprecipitation assays showed that the ternary complex containing Tax-A is more efficiently recruited onto the promoter region of CXCL10, which contains two NF-κB binding sites, than that containing Tax-B.

**Conclusions:**

Our results indicate that different HTLV-1 subgroups are characterized by different patterns of host gene expression. Differential expression of pathogenesis-related genes by subgroup-specific Tax or HBZ may be associated with the onset of HAM/TSP.

**Electronic supplementary material:**

The online version of this article (10.1186/s12977-018-0454-x) contains supplementary material, which is available to authorized users.

## Introduction

Human T-cell leukemia virus type 1 (HTLV-1) was the first human oncogenic retrovirus to be identified [1] and associated with distinct human diseases such as adult T-cell leukemia (ATL) [2, 3] and HTLV-1-associated myelopathy/tropical spastic paraparesis (HAM/TSP) [4, 5]. HTLV-1 can be divided into 7 “subtypes” (subtypes 1a–1g), including 5 African subtypes (subtypes 1b, 1d, 1e, 1f, and 1g), a Melanesian/Australian subtype (subtype 1c), and a cosmopolitan subtype (subtype 1a) based on a phylogenetic analysis of its long terminal repeat (LTR) region. The cosmopolitan subtype has spread worldwide and is further divided into 5 “subgroups” (subgroup A–E of subtype 1a) [6–8]. In the Japanese population, nucleotide sequence variation in the viral transactivator *tax* also determines the HTLV-1 subgroups—namely, “*tax* subgroup-A” and “*tax* subgroup-B” correspond to LTR-based “cosmopolitan subtype 1a subgroup A” and “cosmopolitan subtype 1a subgroup B,” respectively [9]. We therefore refer to “*tax* subgroup-A” and “*tax* subgroup-B” as “subgroup-A” and “subgroup-B” hereafter.

It is well established that both the Tax and HBZ proteins of HTLV-1 transactivate viral and cellular genes and play a key role in HTLV-1 replication and pathogenesis [10–16]. A difference of four nucleotides exists in *tax* and *HBZ* coding regions (i.e., nucleotides 7897, 7959, 8208 and 8344) between subgroup-A Tax (Tax-A) and subgroup-B Tax (Tax-B), which result in two and one amino acid coding changes, respectively, in Tax and HBZ [9]. The most important observation concerning these virus subgroups is that the incidence of HAM/TSP in asymptomatic healthy carriers (HCs) infected with subgroup-A is 2.5 times higher than that in individuals infected with subgroup-B in southern Japan, where both subgroups co-exist [9]. Recently, we reported that this is also the case for inhabitants of Okinawa Prefecture, Japan, which consists of 160 islands and is located in the subtropical southernmost point of Japan [17]. We have also reported that although different HTLV-1 subgroups are characterized by different patterns of *HBZ* and *FoxP3* gene expression in HAM/TSP patients via independent mechanisms of direct transcriptional regulation, these differences do not significantly affect the clinical and laboratory characteristics of HAM/TSP patients [18]. Thus, the mechanism by which HTLV-1 subgroups differ in the risk for HAM/TSP is still largely unknown.

The rationale of this study is that a microarray-based study of subgroup-specific Tax- or HBZ-induced changes of cellular genes would reveal the downstream targets and effectors of these viral transcriptional factors and identify which targets differ between the viral strains. The results will cast light on the causes of HAM/TSP and identify attractive targets for novel therapeutics.

## Methods

### Patients and preparation of clinical samples

This study was approved by the Research Ethics Committee of Kawasaki Medical School (approval number: 1422-3). Written informed consent was obtained from all individuals. Clinical samples from 37 patients with HAM/TSP (19 subgroup-A and 18 subgroup-B infected patients), 20 HCs, and 20 HTLV-1-uninfected normal control subjects (NCs) were analyzed. The diagnosis of HAM/TSP was made according to the World Health Organization diagnostic criteria [19]. The detail information of the patients’ characteristics including proviral load (PVL) was presented in Table 1. Fresh peripheral blood mononuclear cells (PBMCs) were isolated using Histopaque-1077 (Sigma, St. Louis, MO, USA) density gradient centrifugation, washed twice in RPMI medium, and stored in liquid nitrogen as stocked lymphocytes until use.

**Table 1:** Clinical profiles of HTLV-1-associated myelopathy/tropical spastic paraparesis (HAM/TSP) patients

	Tax A (n = 19)	Tax B (n = 18)	*p* value
Age at onset	47.0 ± 16.7	44.2 ± 15.9	0.3467
Sex (Male: Female)	9: 19	14: 70	0.1374
Serum anti-HTLV-1 antibody titer^a^	283.4 ± 461.3	441.4 ± 1049.0	0.6889
HTLV-1 provirus load in PBMCs^b^	873.2 ± 1046.9	745.3 ± 609.2	0.7394
OMDS^c^	5.9 ± 2.1	5.8 ± 2.3	0.6908
Duration of illness	15.1 ± 9.2	17.4 ± 9.8	0.3621

The results represent the mean ± SD

^a^Anti-HTLV-1 antibodies were titrated using the particle agglutination method

^b^HTLV-1 Tax copy number per 10^4^ PBMCs

^c^OMDS: Osame Motor Disability Score (see Additional file 1: Table S1)

### Cells

Twelve HTLV-1-infected human T-cell lines (MT2, MT4, C5MJ, MT1, ATL43Tb, ATL55T, ED, TLOm1, ILT-M1, HCT1, HCT4, and HCT5) and three HTLV-1-uninfected T-cell lines (Jurkat, CEM, and Molt4) were used in this study. MT2, MT4, and C5MJ are chronically HTLV-1-infected cell lines derived from cord blood mononuclear cells exposed to HTLV-1 from patients with ATL, i.e., HTLV-1-transformed T-cell lines. MT1, ATL43Tb, ATL55T, ED, and TLOm1 are HTLV-1-infected cell lines derived from patients with ATL. Among these ATL cell lines, only ATL55T is IL-2-dependent. ILT-M1, HCT1, HCT4, and HCT5 are IL-2-dependent HTLV-1-infected T-cell lines derived from patients with HAM/TSP. The Tax-inducible JPX-9 cell line was derived from the Jurkat HTLV-1-negative human T-cell leukemia cell line and expresses biologically active Tax protein under the control of the metallothionein promoter [20]. These cells were cultured in RPMI 1640 medium supplemented with 10% heat-inactivated fetal calf serum (FCS), 50 U/ml penicillin, and 50 µg/ml streptomycin (Wako Pure Chemical, Osaka, Japan) at 37 °C and 5% CO_2_. For IL-2-dependent cell lines, 10 U/ml (for ATL55T, HCT1, HCT4, and HCT5) or 30 U/ml (for ILT-M1) of recombinant human IL-2 (Wako) were added to the culture. Jurkat Tet-On 3G (Takara Bio USA, Mountain View, CA) is a Jurkat-derived T-cell line made for use in the Tet-On 3G Inducible Gene Expression System (Takara Bio USA). Jurkat Tet-On 3G cells were cultured in RPMI1640 supplemented with 10% FCS, 100 units/ml penicillin, 100 µg/ml streptomycin (Wako), and 200 µg/ml G418 (SIGMA-ALDRICH Japan, Tokyo, Japan) at 37 °C in 5% CO_2_. Human embryonic kidney 293T cells were grown in Dulbecco’s modified Eagle’s medium (DMEM) supplemented with 4 mM glutamine, 10% FCS, 100 units/ml penicillin, and 100 µg/ml streptomycin at 37 °C and 5% CO_2_.

### Plasmid construction

All primers used for the plasmid construction are listed in Additional file 2: Table S2. The following pTRE3G-based plasmids were constructed for inducible expression in the presence of doxycycline (Dox) (Takara Bio USA) in Jurkat Tet-On 3G cells. To construct pTRE3G-Tax-A or pTRE3G-Tax-B, DNA fragments corresponding to the Tax coding sequence were amplified via PCR, using Tax-FOR and Tax-REV as primers and pCG-Tax-A or pCG-Tax-B [18] as a template. PCR products were digested with SalI and BglII, then cloned into SalI- and BamHI-digested pTRE3G-IRES (Takara Bio USA). To construct pTRE3G-HBZ-A or pTRE3G-HBZ-B, DNA fragments corresponding to the HBZ coding sequence were amplified via PCR, using HBZ-FOR and HBZ-REV as primers and pCMV-HA-HBZ-A or pCMV-HA-HBZ-B [18] as the template. PCR products were digested with SalI and BglII, then cloned into SalI- and BglII-digested pTRE3G-IRES.

The following pCAGGS-P7- and pGL3-based plasmids were constructed for the immunoprecipitation assay and luciferase assay. To construct pCAGGS-P7-Tax-A and pCAGGS-P7-Tax-B, DNA fragments corresponding to the Tax coding sequence were amplified by PCR using Tax-FOR and Tax-Stop-REV as primers and pCG-Tax-A or pCG-Tax-B [18] as a template. PCR products were phosphorylated with T4 polynucleotide kinase and digested with SalI and cloned into SalI- and EcoRV-digested pCAGGS-P7.

To construct pCAGGS-P7-Tax-A-FLAG, pCAGGS-P7-Tax-B-FLAG, and pCAGGS-P7-Tax-1 (225-232)-FLAG, DNA fragments corresponding to the Tax coding sequence were amplified via 1st-PCR using Tax-FOR and Tax-FLAG-REV as primers and pCAGGS-P7-Tax-A, pCAGGS-P7-Tax-B, or CSII-EF-Tax-1(225-232)-FLAG [21] as the templates. To add a tandem FLAG-tag sequence to the C-terminus of Tax proteins, DNA fragments were amplified via 2nd-PCR using Tax-FOR and FLAG-Tandem-REV as primers and the 1st-PCR products as templates. Next, to fuse the NotI-recognized DNA sequence at the 3′ site of the FLAG-tag, DNA fragments were amplified via 3rd-PCR using Tax-FOR and FLAG-Tandem-NotI-REV as primers and the 2nd-PCR products as templates. The 3rd-PCR products were digested with SalI and NotI, and cloned into SalI- and NotI-digested pCAGGS-P7. To construct pCAGGS-P7-Tax-2B-FLAG, DNA fragments were amplified via PCR using Tax-2-FOR and Tax-2-FLAG-Tandem-REV as primers and CSII-EF-Tax-2-FLAG [21] as the template. To add a tandem FLAG-tag sequence to the C-terminus of Tax proteins, DNA fragments were amplified via 2nd-PCR using Tax-2-FOR and FLAG-Tandem-REV as primers and the 1st-PCR products as templates. Next, to fuse the NotI-recognized DNA sequence to the 3′ site of the FLAG-tag, DNA fragments were amplified via 3rd-PCR using Tax-2-FOR and FLAG-Tandem-*NotI*-REV as primers and the 2nd-PCR products as templates. The 3rd-PCR products were digested with SalI and NotI, and cloned into SalI- and NotI-digested pCAGGS-P7.

To amplify the human C-X-C motif chemokine 10 (CXCL10) promoter region, harboring a sequence spanning nucleotide positions − 875 to + 97 (where the transcription start site is set to be + 1) or − 279 to + 97 [22], PCR was carried out using CXCL10-875-FOR or CXCL10-279-FOR and CXCL10-REV as primer sets and genomic DNA derived from Jurkat cells as templates. PCR products were digested with KpnI and XhoI and cloned into KpnI- and XhoI-digested pGL3-Basic (Promega Corporation, Madison, WI). The resultant plasmid was designated pGL3-CXCL10-Long-Luc or pGL3-CXCL10-Short-Luc. Mutations in the NF-κB1 (5′-GGGACTTCCC-3′), NF-κB2 (5′-GGGAAATTCC-3′), and AP-1 (5′-TAAGTCA-3′) binding sites in the CXCL10 promoter were generated using overlapping-PCR using the primers listed in Additional file 2: Table S2. To construct pGL3-CXCL10 (κB1-mut)-Luc (κB1 mutant sequence: 5′-GTGACTTCAC-3′), two DNA fragments corresponding to the CXCL10 promoter sequence were amplified via PCR using CXCL10-279-FOR and κB1-mut-REV or CXCL10-REV and κB1-mut-FOR as primers and pGL3-CXCL10-Short-Luc as the PCR template. Full-length CXCL10 (κB1-mut) DNA fragment was amplified via PCR using CXCL10-279-FOR and CXCL10-REV as primers. PCR products were digested with KpnI and XhoI and cloned into the KpnI- and XhoI-digested pGL3-Basic. Likewise, pGL3-CXCL10 (κB2-mut)-Luc (κB2 mutant sequence: 5′-GTGAAATTAC-3′), pGL3-CXCL10 (κB1 + κB2-mut)-Luc (mutation of both the κB1 and κB2), and pGL3-CXCL10 (AP-1-mut)-Luc (AP-1 mutant sequence: 5′-TAAGAGA-3′) were constructed using primers listed in Additional file 2: Table S2.

The sequences of all recombinant plasmids were confirmed using Sanger sequencing.

### Transfection by electroporation

DNA electroporation was performed with the Neon™ Transfection System (Invitrogen, Carlsbad, CA). For the electroporation, Jurkat and Jurkat Tet-On 3G cells were grown in non-treated cell culture dishes and transferred into a 15 ml polypropylene tube. Cells were centrifuged at 700×*g* for 3 min. The pellet was re-suspended in 10 ml of PBS, and cells were counted. Cells were pelleted again and re-suspended in Buffer R (included with Neon™ Kits) to a final concentration of 2.0 × 10^7^/ml. 100 µl or 10 µl of cell suspension containing 2.0 × 10^6^ cells or 2.0 × 10^5^ cells, respectively, and 10 µg or 3 µg of DNA plasmid were transferred into the Neon™ tip (100 µl tip or 10 µl tip). The electroporation was carried out under appropriate conditions as per the manufacturer’s instructions. After 24 h transfection, Tax-A, Tax-B, HBZ-A, or HBZ-B proteins were induced in Jurkat Tet-On 3G cells by adding Dox (final concentration: 3 ng/ml) for 24 h. Uninduced Jurkat Tet-On 3G cells were used as a baseline reference.

### Western blotting

Whole-cell lysates were subjected to SDS–polyacrylamide gel electrophoresis (SDS-PAGE) and transferred to polyvinylidene difluoride (PVDF) membranes (pore size 0.45 µm, Merck Millipore, MA, USA). PVDF membranes were blocked with 5% skim milk or Blocking One (Nacalai tesque, Kyoto Japan) in Tris-buffered saline containing 0.1% Tween 20 (TBS-T) and probed with anti-Tax mouse monoclonal (Lt-4), anti-HBZ mouse monoclonal (#7-1) [23], anti-HBZ rat monoclonal (#4B12), anti-β-actin rabbit polyclonal (PM053, MEDICAL & BIOLOGICAL LABORATORIES, Nagoya, Japan), or anti-α-tubulin rabbit polyclonal (PM054, MEDICAL & BIOLOGICAL LABORATORIES) antibodies. PVDF membranes were washed with TBS-T and incubated with IRDye 680RD Goat anti-Mouse IgG, IRDye 800CW goat anti-rat IgG, or IRDye 800CW goat anti-rabbit IgG (LI-COR Biosciences, Lincoln, NE). After washing with TBS-T, the proteins were detected using Odyssey CLx Infrared Imaging System (LI-COR Biosciences).

### Microarray analysis

The microarray experiments were performed in triplicate, and data are shown as mean values.

### RNA processing and labeling

After 24 h of cultivation, the transfected cells were harvested and processed for subsequent analysis. RNA was prepared using the RNAeasy Mini Kit column purification (QIAGEN, Tokyo, Japan) following the manufacturer’s instructions. RNA was quantified using a NanoVue™ spectrophotometer (GE Healthcare Japan, Tokyo, Japan) and quality was monitored using 1% Agarose/Formaldehyde gel electrophoresis. Cyanine-3 (Cy3) labeled cRNA was prepared from 0.1 µg RNA using the One-Color Low Input Quick Amp Labeling Kit (Agilent Technologies, Tokyo, Japan) according to the manufacturer’s instructions, followed by RNAeasy Mini Kit column purification (QIAGEN). Dye incorporation and cRNA yield were assessed using the NanoDrop™ spectrophotometer (Thermo Fisher Scientific) and the Agilent 2100 Bioanalyzer (Agilent).

### cRNA hybridization and scanning

600 ng of Cy3-labelled cRNA (specific activity > 6.0 pmol Cy3/µg cRNA) was fragmented at 60 °C for 30 min in a reaction volume of 25 µl containing 1 × Agilent fragmentation buffer and 2 × Agilent blocking agent following the manufacturer’s instructions. On completion of the fragmentation reaction, 25 µl of 2 × GE Agilent hybridization buffer, HI-RPM was added to the fragmentation mixture and hybridized to Agilent SurePrint G3 Human GE 8 × 60 K V2 Microarrays (Catalog Code: G4851B) and V3 Microarrays (G4851C) (Agilent) for 17 h at 65 °C in a rotating Agilent hybridization oven. After hybridization, microarrays were washed for 1 min at room temperature with GE Wash Buffer 1 (Agilent) and 1 min with 37 °C GE Wash buffer 2 (Agilent), then dried immediately by brief centrifugation. Slides were scanned immediately after washing on the Agilent DNA Microarray Scanner (G2505C) (Agilent) using one color scan setting for 8 × 60 k array slides (Scan Area 61 × 21.6 mm, Scan resolution 3 µm, dye channel set to Green).

The scanned images were analyzed using Feature Extraction Software 11.0.1.1 (Agilent) at default parameters (protocol GE1-1100_Jul11 and Grid: 039494_D_F_20140813) to obtain background subtracted and spatially detrended Processed Signal intensities. Features flagged in Feature Extraction as Feature Non-uniform outliers were excluded. Data were analyzed using GeneSpring GX (Agilent). Microarray data have been deposited in the NCBI Gene Expression Omnibus (www.ncbi.nlm.nih.gov/geo/) (GEO ID: GSE103323 and GSE121201). To detect differentially expressed genes, we applied Significance Analysis of Microarrays (SAM) with R package ‘samr’. In the SAM analysis, we used two-class unpaired comparison with a threshold of q-value < 0.05 between control and Tax-A, control and Tax-B, control and HBZ-A, control and HBZ-B. When a gene has more than two probes, fold-change in the gene is defined to be the average fold-changes in multiple probes assigned to be the gene.

### Genomic DNA and RNA extraction and cDNA synthesis

Genomic DNA was extracted from PBMCs using the QIAamp Blood Kit (Qiagen, Tokyo, Japan). RNA was extracted from PBMCs using the RNeasy Mini Kit with on-column DNase digestion (Qiagen). cDNA was synthesized using the PrimeScript^®^ RT Reagent Kit (Takara, Kyoto, Japan). All reaction procedures were performed as suggested by the manufacturer.

### Quantification of HTLV-1 proviral load and anti-HTLV-1 antibody titers

To examine the HTLV-1 proviral load (PVL), quantitative PCR was performed using 100 ng of genomic DNA (roughly equivalent to 10^4^ cells) from PBMCs as previously reported [24]. The amount of HTLV-1 proviral DNA was determined using the following formula: copy number of HTLV-1 *tax* per 1 × 10^4^ PBMCs = [(copy number of tax)/(copy number of β − actin/2)] × 10^4^. All samples were examined in triplicate. Antibody titers to HTLV-1 were determined using a particle agglutination method (Serodia-HTLV-1^®^; Fujirebio, Tokyo, Japan), according to the manufacturer’s instructions.

### Real-time quantitative reverse transcription PCR analysis

To estimate *tax* and *HBZ* mRNA expression levels, a real-time quantitative reverse transcription PCR (real-time qRT-PCR) was performed as previously reported [14]. Human *CXCL10* (Applied Biosystems Hs01085834_m1) gene specific assays were used for *CXCL10* quantifications. The expression levels of these genes were normalized to the expression levels of human hypoxanthine phosphoribosyltransferase 1 (*HPRT1*) (Human HPRT1 Endogenous Control 4333768; Applied Biosystems, Foster City, CA). All assays were performed in triplicate.

### ELISA

Human CXCL10 levels were determined using ELISA, employing the Quantikine^®^ ELISA Human CXCL10/IP-10 Immunoassay kit (R&D Systems, Minneapolis, MN) according to the manufacturer’s instruction.

### Luciferase assay

To compare CXCL10 promoter activity, Jurkat cells (2.0 × 10^5^ cells) were transfected with 1.5 µg of firefly luciferase reporter plasmid, i.e., pGL3-CXCL10-Long-Luc, pGL3-CXCL10-Short-Luc, pGL3-CXCL10 (κB1-mut)-Luc, pGL3-CXCL10 (κB2-mut)-Luc, pGL3-CXCL10 (κB1 + κB2-mut)-Luc, or pGL3-CXCL10 (AP-1-mut)-Luc, 1.5 µg of Tax protein expression plasmid pCAGGS-P7-Tax-A-FLAG or pCAGGS-P7-Tax-B-FLAG), and 250 ng of a *Renilla* reporter plasmid (pRL-RSV) per well by electroporation. The reporter construct containing a luciferase gene fused to five repeats of the NF-κB site of the IL-2Ra gene [25] was tested with same procedure. We also tested CXCL10 promoter activity using 293T cells. The reporter plasmid pGL3-CXCL10-Long-Luc or pGL3-CXCL10-Short-Luc, 1.5 µg of Tax protein expression plasmid (pCG-Tax-A or pCG-Tax-B), and 250 ng of a *Renilla* reporter plasmid (pRL-RSV) were transfected into 293T cells (2.0 × 10^5^ cells) per well by using Lipofectamine LTX with PLUS reagent (Invitrogen, Carlsbad, CA, USA) according to the manufacturer’s instructions. After 24 h transfection, cells were harvested and lysed, and reporter activities were measured using the Dual-Luciferase Reporter Assay System (Promega, Tokyo, Japan). Each experiment was performed in triplicate, and the data represent the mean ± SD of three independent experiments. Firefly Luciferase values were normalized to values of the *Renilla* reporter.

### Immunoprecipitation assay

Jurkat cells (2.0 × 10^6^ cells) were transfected with 10 µg of each indicated plasmid, i.e., pCAGGS-P7-empty, pCAGGS-P7-Tax-A-FLAG, pCAGGS-P7-Tax-B-FLAG, pCAGGS-P7-Tax-1(225-232)-FLAG, and pCAGGS-P7-Tax-2B-FLAG, by electroporation and then cultured for 24 h at 37 °C. The harvested cells were solubilized using a lysis buffer (20 mM Tris–HCl [pH 7.9], 100 mM NaCl, 0.1% Triton X-100). After sonication, homogenates were centrifuged at 14,000×*g* at 4 °C for 5 min, and the supernatant fraction was used as extracts for immunoprecipitation. Then, Tax protein was immunoprecipitated with anti-FLAG gels. The cell extracts were incubated with ANTI-FLAG M2 Affinity Gel (SIGMA-ALDRICH Japan, Tokyo, Japan) at 4 °C for 1 h. After incubation, the resins were collected via brief centrifugation and washed two times with the lysis buffer. The resin-bound proteins were eluted by boiling in the SDS-PAGE Sample Loading Buffer (Takara Bio USA) and subjected to 10% SDS-PAGE, followed by western blotting using anti-FLAG M2 (SIGMA-ALDRICH), anti-p105/p50 (#3035: Cell Signaling Technology Japan, Tokyo, Japan), anti-p100/p52 (#4882: Cell Signaling Technology), anti-p65 (#4764: Cell Signaling Technology), anti-RelB (#4922: Cell Signaling Technology), and anti-c-Rel (#4727: Cell Signaling Technology) antibodies. An aliquot of the cell lysates removed before immunoprecipitation was also characterized as an input (Input). The amounts of p100, p52, RelB, p65, p105, p50, and c-Rel proteins in immunoprecipitated samples were quantified by the densitometry scanning of corresponding bands of the western blot using Image J software.

### Chromatin immunoprecipitation (ChIP) assay

Jurkat cells (2.0 × 10^6^ cells) were co-transfected with 9 µg of each indicated plasmid, i.e., pCAGGS-P7-empty, pCAGGS-P7-Tax-A-FLAG, and pCAGGS-P7-Tax-B-FLAG, and 1 µg of pEF-321T (plasmid encoding SV40 T antigen for amplification of transfected pCAGGS-P7) by electroporation and then cultured for 24 h at 37 °C. Transfected cells were treated with 1% formaldehyde at room temperature for 15 min. Fixation was quenched by the addition of glycine at the final concentration of 125 mM, then cells were washed twice with PBS and collected by centrifugation. Cell pellets were resuspended in ChIP lysis buffer (50 mM Tris–HCl [pH 7.9], 1% Triton X-100, 0.1% SDS, and 10 mM EDTA). Cell lysates were sonicated to shear the chromatin DNA to ~ 500 base-pairs in size and then diluted with four volumes of ChIP dilution buffer (12.5 mM Tris–HCl [pH 7.9], 187.5 mM NaCl, and 1% Triton X-100). Lysates clarified by centrifugation were incubated with anti-Lt-4 antibody at 4 °C overnight. Antibody-protein-DNA complexes were incubated with Salmon Sperm DNA/Protein A Agarose (MILLIPORE, Temecula CA), and immunoprecipitates collected onto Protein A Agarose were washed with a high salt wash buffer (20 mM Tris–HCl [pH 7.9], 500 mM NaCl, 2 mM EDTA, 0.1% SDS, and 1% Triton X-100), a low salt wash buffer (20 mM Tris–HCl[pH 7.9], 100 mM NaCl, 2 mM EDTA, 0.1% SDS, and 1% Triton X-100), LiCl buffer (10 mM Tris–HCl[pH 7.9], 250 mM LiCl, 1% NP-40, and 1 mM EDTA), and twice with TE (10 mM Tris–HCl[pH 7.9] and 1 mM EDTA) successively. Then, bound proteins were eluted from the agarose beads in elution buffer (1% SDS and 100 mM NaHCO_3_) by incubation at room temperature for 15 min. Crosslinking was reversed by incubation at 65 °C overnight. All samples were treated with 45 μg/ml of proteinase K (Nacalai tesque, Kyoto, Japan) at 50 °C for 2 h, then 40 µg of glycogen (Nacalai tesque) was added, followed by extraction with phenol:chloroform:isoamyl alcohol and precipitation with ethanol. DNA fragments were subjected to PCR or quantitative real-time PCR using EmeraldAmp Master Mix (TaKaRa, Shiga, Japan) or SYBR Premix EX Taq II (TaKaRa, Shiga, Japan) with specific primer sets: 5′-GAA AGT GAA ACC TAA TTC ACT ATT ACC AA-3′ and 5′-ACT TAG CAA AAC CTG CTG GCT GTT-3′ for genomic DNA fragment containing both NF-κB and AP-1 binding sites. Three independent experiments were performed, and the data represent the mean ± SD.

### Statistical analysis

To test for significant differences among four different groups of subjects (HAM/TSP patients with subgroup-A, HAM/TSP patients with subgroup-B, HCs and NCs), the data were statistically analyzed using one-way analysis of variance (ANOVA). Inter-group comparisons were done by Scheffe’s post hoc multiple comparisons test. The Mann–Whitney U test was used to compare data between two groups (i.e., difference between Tax-A and Tax-B with respect to transcriptional activity for both the short and long CXCL10 promoters, see Fig. 6b). Correlations between variables were examined using Spearman rank correlation analyses. The results shown are the mean ± SD where applicable. Results were considered statistically significant at *p* values < 0.05.

## Results

### Expression of each subgroup Tax or HBZ protein under the control of an inducible promoter revealed different target gene profiles

**Fig. 1:**
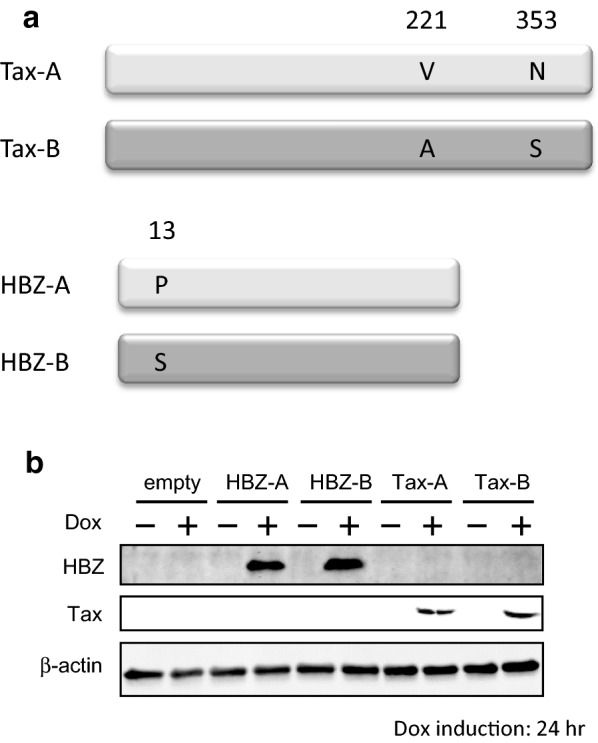
Expression of each subgroup Tax or HBZ protein under transcriptional control of a doxycycline (Dox)-inducible promoter. **a** Schematic representation of the subgroup-specific Tax and HBZ proteins. Proteins are shown as grey bars, and differences in amino acid sequences between subgroup-specific Tax, i.e., Tax-A and Tax-B, or HBZ, i.e., HBZ-A and HBZ-B, proteins are indicated by a single letter amino acid abbreviation. **b** The Dox-induced expression of each Tax or HBZ protein is shown. Western blot analysis confirmed that each subgroup-specific Tax and HBZ protein was appropriately induced following Dox treatment

The differences in amino acid sequences between subgroup-specific Tax, i.e., Tax-A and Tax-B, or HBZ, i.e., HBZ-A and HBZ-B, proteins are depicted in Fig. 1a. Protein expression in Jurkat Tet-On 3G cells was induced by adding Dox to culture media at a final concentration of 3 ng/ml for 24 h at 37 °C. Western blot analysis confirmed that each subgroup-specific Tax and HBZ protein was appropriately induced following Dox treatment (Fig. 1b). Microarray was performed on RNAs derived from these samples.

**Fig. 2:**
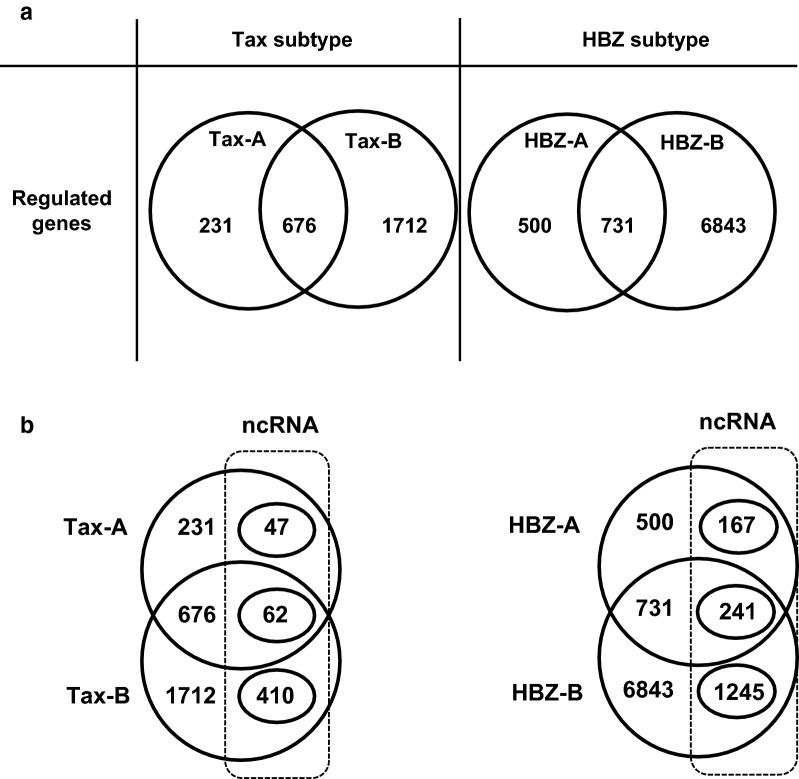
Results of cDNA microarrays to examine subgroup-specific Tax or HBZ-mediated transcriptional changes in Jurkat Tet-On human T-cells. After transfection, Tax-A, Tax-B, HBZ-A or HBZ-B proteins were induced in Jurkat Tet-On 3G cells by adding Dox (final concentration: 3 ng/ml) for 24 h. Uninduced Jurkat Tet-On 3G cells were used as a baseline reference. Microarray was performed on RNAs derived from these samples. **a** The numbers of genes that occurred in each cluster are shown. We identified 231, 676, and 1712 genes that were specifically up- or down-regulated by Tax-A, by both Tax-A and Tax-B, and by Tax-B, respectively. In HBZ, 500, 731, and 6843 genes were specifically up- or down-regulated by HBZ-A, by both HBZ-A and HBZ-B, and by HBZ-B respectively. **b** Among 231, 676, and 1712 genes were specifically upregulated by HBZ-A, by both HBZ-A and HBZ-B, and by HBZ-B, respectively, 47, 62, and 410 genes were ncRNAs. Similarly, among 500, 731, and 6843 genes that were specifically upregulated by Tax-A, by both Tax-A and Tax-B, and by Tax-B, respectively, 167, 241, and 1245 genes were ncRNAs

The results of microarray analysis are shown in Fig. 2. In our microarray analysis, genes were considered differentially expressed on the significant level using SAM analysis (q-value < 0.05). The numbers of genes that occurred in each cluster are shown in Fig. 2a; we identified 231, 676, and 1712 genes specifically regulated by Tax-A, by both Tax-A and Tax-B, and by Tax-B, respectively. In HBZ, 500, 731, and 6843 genes were specifically regulated by HBZ-A, by both HBZ-A and HBZ-B, and by HBZ-B respectively. These data indicated that the number of genes regulated by HBZ was 2–3 times higher than that regulated by Tax.

Next, to define the gene expression profiles of each subgroup-specific Tax or HBZ, we manually created a list of the top 50 genes up-regulated by each subgroup of Tax, i.e., Tax-A and -B) (Table 2), or HBZ, i.e., HBZ-A and -B (Table 3). The data show the average fold-change expression in each subgroup-specific Tax (Table 2) or HBZ (Table 3) in Dox-treated Jurkat Tet-On 3G cells, compared to untreated cells. Gene expression levels were represented with red (high expression) or blue (low expression) in each block. The genes are ranked in order of their average fold-change expression of Tax-A, and the next column represents the average fold-change expression of Tax-B. Interestingly, as shown in Table 2, all of the top 50 genes regulated by Tax were more strongly upregulated by Tax-A than Tax-B (Table 2). In contrast, as shown in Table 3, some target genes were preferentially induced by HBZ-A, rather than by HBZ-B, whereas other target genes were preferentially induced by HBZ-B, rather than by HBZ-A. There were also some target genes regulated by both subtypes HBZ at similar efficiency. These results indicate that the Tax and HBZ molecule of each respective subgroup has a different target gene profile. Importantly, some of the genes listed in Table 2 (shown in red letters), such as EBI3 (= Epstein-Barr virus-induced gene 3, a component of IL-27) [26], VCAM-1 [27], IL-13 [28], and CCL1 [29] have already been identified as Tax target genes in previous studies, validating our gene induction system. It is also noteworthy that among the Tax- or HBZ-regulated genes, there were a set of ncRNAs, including miRNAs and long non-coding RNAs (lncRNAs) (Fig. 2b and Tables 2, 3).

**Table 2:**
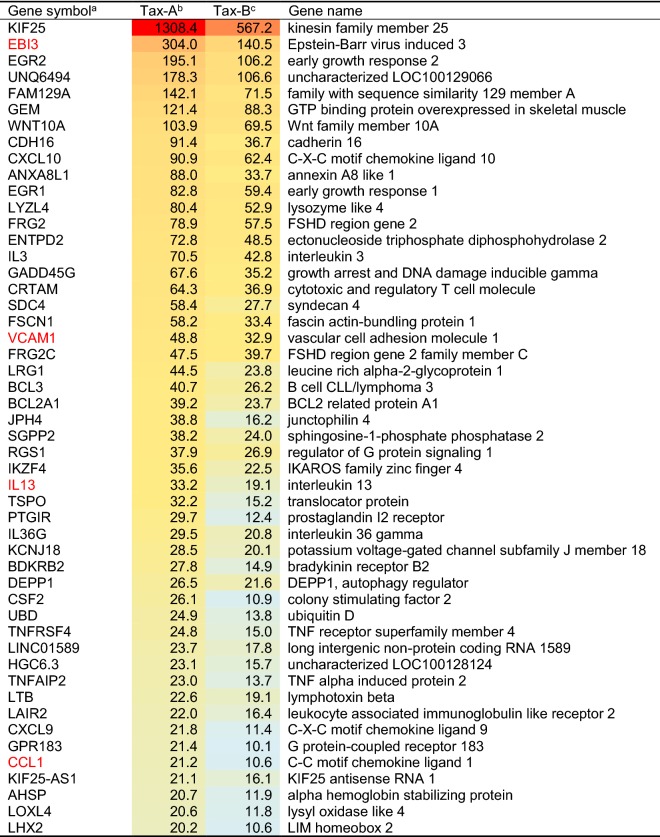
Top 50 up-regulated genes in Tax-induced Jurkat Tet-On 3G cells

^a^Genes were ranked in order of fold change by Tax-A induction

^b,c^In order to identify up-regulated genes in Tax-A or Tax-B induction, cells transfected with pTRE3G-empty were used as normalization samples. The microarray experiments were performed in triplicate, and data were shown as mean values

**Table 3:**
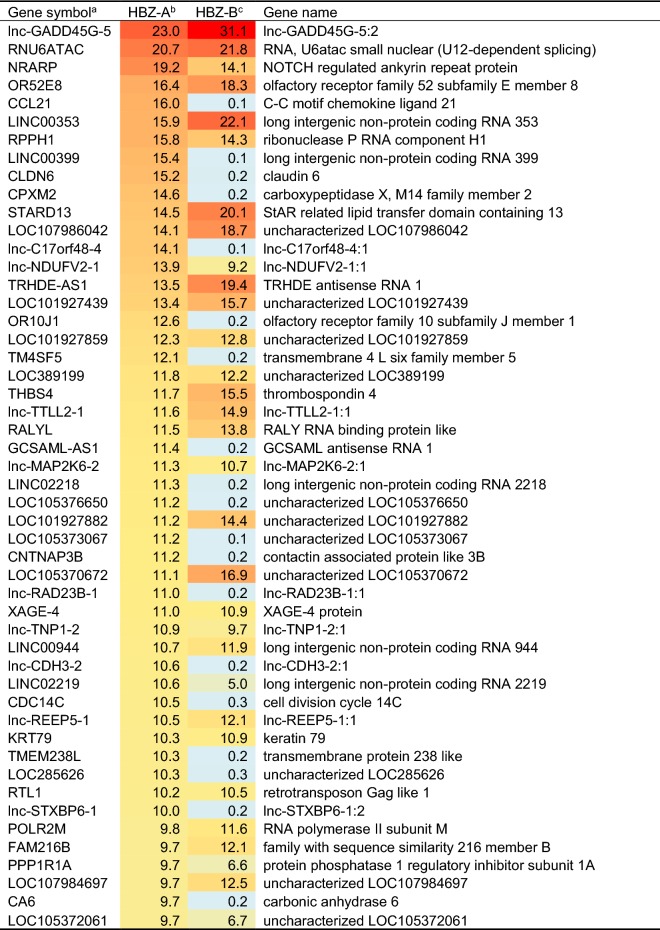
Top 50 up-regulated genes in HBZ-induced Jurkat Tet-On 3G cells

^a^Genes were ranked in order of fold change by HBZ-A induction

^b,c^In order to identify up-regulated genes in HBZ-A or HBZ-B induction, cells transfected with pTRE3G-empty were used as normalization samples. The microarray experiments were performed in triplicate, and data were shown as mean values

### Validation of differentially expressed genes identified by microarray using qRT-PCR in PBMCs of HAM/TSP patients with HTLV-1 subgroup A or B

**Fig. 3:**
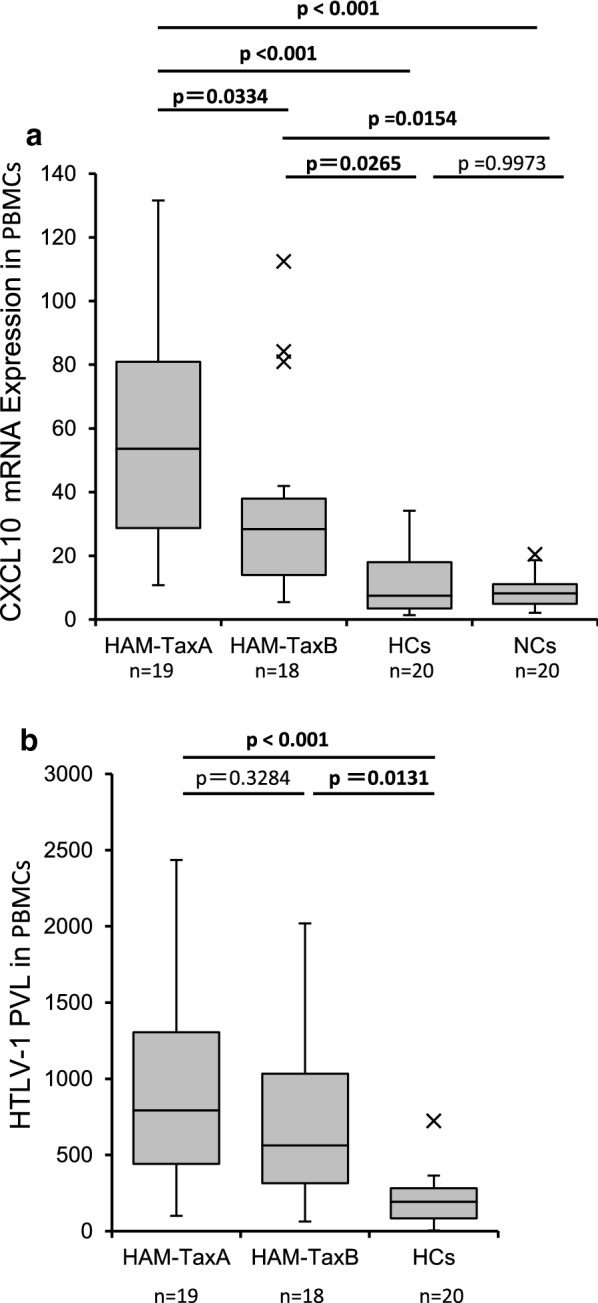
Validation of differentially expressed genes identified by microarray using qRT-PCR in PBMCs of HAM/TSP patients with HTLV-1 subgroup (**a**) or (**b**). To confirm whether the obtained in vitro gene expression profiles have clinical significance, we performed real-time qRT-PCR on PBMCs of 37 patients with HAM/TSP (19 subgroup-A and 18 subgroup-B), 20 HCs, and 20 NCs. Among identified Tax-responsive genes, we measured the expression levels of *CXCL10* mRNA levels. **a** The expression levels of *CXCL10* in HAM/TSP patients with subgroup-A was significantly higher than in HAM/TSP patients with subgroup-B (p = 0.0334 by Scheffe’s post hoc multiple comparisons test). **b** There was no significant difference in HTLV-1 PVL between HAM/TSP patients with subgroup-A or -B

To confirm whether the obtained in vitro gene expression profile had clinical significance, we performed real-time qRT-PCR on the PBMCs of 37 patients with HAM/TSP (19 subgroup-A and 18 subgroup-B), 20 HCs, and 20 NCs (Fig. 3). Among the identified Tax-responsive genes, we measured the expression levels of *CXCL10* mRNA, since *CXCL10* was preferentially induced by Tax-A rather than by Tax-B (Table 2). In accordance with the microarray data, the results showed that the expression levels of *CXCL10* in HAM/TSP patients with subgroup-A were significantly higher than in HAM/TSP patients with subgroup-B (*p* = 0.0334 by Scheffe’s post hoc multiple comparisons test.) (Fig. 3a). Meanwhile, there was no significant difference in HTLV-1 PVL between HAM/TSP patients with subgroup-A or -B (Fig. 3b).

### More efficient induction of chemokine CXCL10 by Tax-A than by Tax-B

**Fig. 4:**
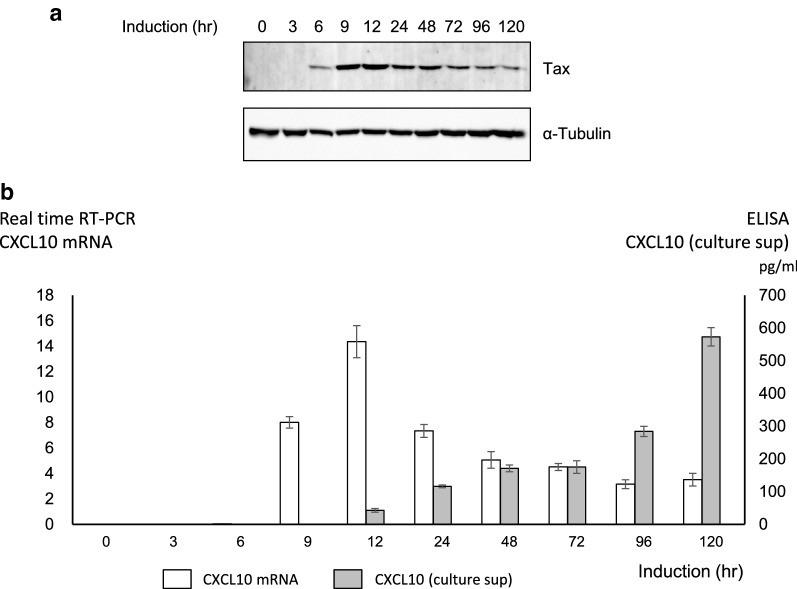
CXCL10 expression is induced along with Tax in JPX-9 cells. **a** Western blot analysis of Tax expression in JPX-9 cells treated with 10 μM CdCl_2_. Cell lysates were prepared from CdCl_2_-treated JPX-9 cells at the indicated time points, and Tax expression was confirmed by western blotting with Lt-4 anti-Tax monoclonal antibody. Equal sample loading was verified with anti-α-Tubulin (bottom). Tax protein was almost undetectable in JPX-9 before the induction, but became detectable 6 h after the addition of CdCl_2_ to the culture medium. **b** Induction of CXCL10 mRNA and protein expression in JPX-9 cells treated with CdCl_2_. CXCL10 mRNA and protein levels after Tax induction were detected by quantitative real-time PCR and ELISA, respectively. The copy number of CXCL10 mRNA was normalized by the copy number of hypoxanthine guanine phosphoribosyl transferase 1 (HPRT1) mRNA. Quantitative real-time PCR results indicate that CXCL10 mRNA expression was induced along with Tax protein expression in JPX-9 cells (white bars). ELISA data indicated that CXCL10 protein became detectable 12 h after the addition of CdCl_2_ to the culture medium, i.e. 6 h after detection of Tax protein and 3 h after detection of CXCL10 mRNA, and the CXCL10 protein levels were still increasing even at 120 h after the addition of CdCl_2_ (gray bars). The data are representative of 3 independent experiments. Data shown as mean ± SD, n  =  3

Among the genes differentially induced by Tax-A and Tax-B (Table 2), we focused our attention especially on the chemokine genes (Table 4), particularly because chemokines have been considered to play relevant roles in the pathogenesis of HAM/TSP [30, 31], and both CXCL9 [32] and CXCL10 [33] (listed in Table 2) have already been identified as Tax target genes in previous studies, providing further validation of our gene induction system. In particular, CXCL10 has been proposed as the most viable, prognostic biomarker for HAM/TSP, as the cerebrospinal fluid (CSF) levels of CXCL10 were well correlated with disease progression of HAM/TSP, better even than HTLV-1 PVL in PBMCs, i.e., the number of HTLV-1-infected cells [31, 32]. Our data demonstrated that CXCL10 was more efficiently upregulated by Tax-A, which is associated with an increased risk of developing HAM/TSP, than by Tax-B (Table 2), suggesting that the HTLV-1 subgroups are associated with changes in host gene expression closely associated with HAM/TSP pathogenesis. To further validate the results using a different inducible protein expression system, we used JPX-9 cells [20], a subclone of Jurkat generated by stable transfection of a functional Tax expression-plasmid vector, and induced Tax expression by adding cadmium chloride (CdCl_2_) to the culture medium (10 µM final concentration). Tax protein was almost undetectable in JPX-9 before the induction but became detectable 6 h after the addition of CdCl_2_ to the culture medium (Fig. 4a). Quantitative real-time PCR results indicate that CXCL10 mRNA expression was induced along with Tax protein expression in JPX-9 cells (Fig. 4b, white bars). ELISA data indicated that CXCL10 protein became detectable 12 h after the addition of CdCl_2_ to the culture medium, i.e. 6 h after detection of Tax protein and 3 h after detection of CXCL10 mRNA (Fig. 4b, gray bars), and the CXCL10 protein levels were still increasing even 120 h after the addition of CdCl_2_.

**Table 4:** Chemokine genes differentially induced by Tax-A and Tax-B

	Fold change					
	Tax-A	Tax-B	Tax A/B ratio	Gene name	Partner (receptor or ligand)	References
Chemokine	90.9	62.4	1.5	CXCL10	CXCR3	[32, 45, 46]
	21.8	11.4	1.9	CXCL9	CXCR3	[32, 46]
	N.D.^a^	2.4		CCL22	CCR4	[47]
	21.2	10.6	2.0	CCL1	CCR8	[29, 48]
	N.D.	2.9		CCL5	CCR1, CCR3, CCR5	[49, 50]
	5.8	2.3	2.5	CCL20	CCR6	[51]
	9.0	6.0	1.5	XCL1	XCR1	[52]
Chemokine receptor	5.6	3.8	1.5	CXCR6	CXCL16	None reported
	2.2	2.2	1.0	CCR7	CCL19, CCL21	[53]
	1.5	N.D.		CCRL2/CCR11	CCL2, CCL5, CCL7, CCL8	None reported

^a^Not detected

### Preferential expression of CXCL10 in HTLV-1-infected T-cell lines derived from patients with ATL and HAM/TSP

**Fig. 5:**
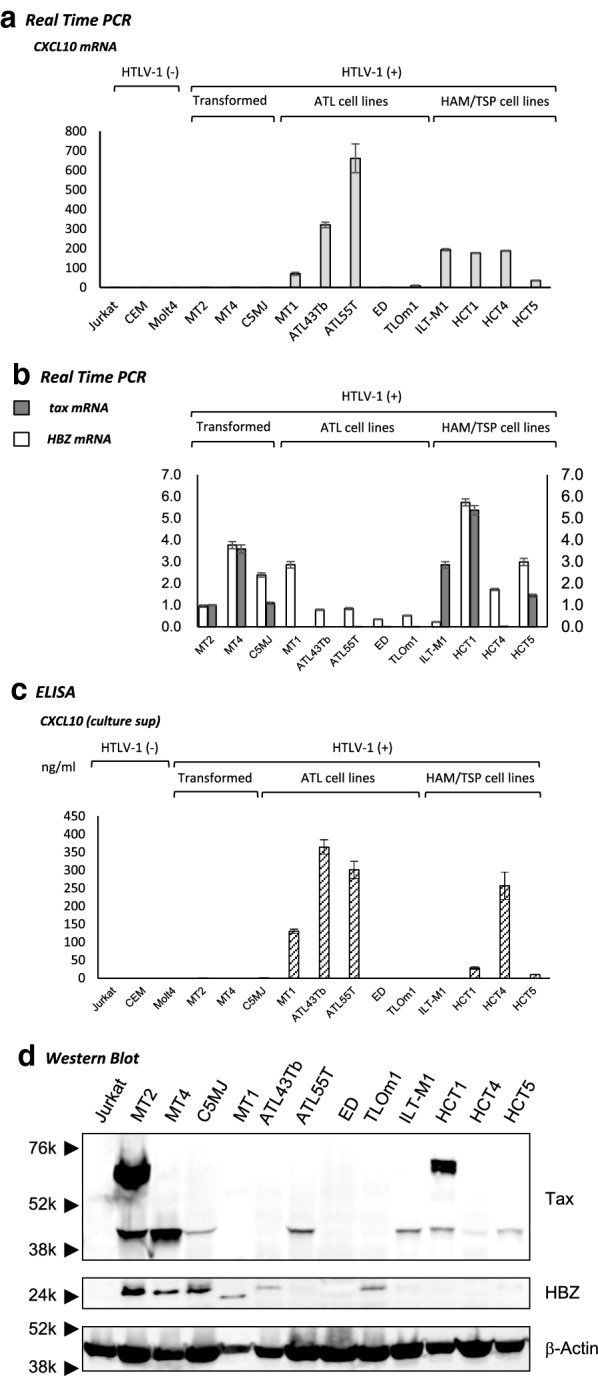
Preferential expression of CXCL10 in Human T-cell leukemia virus type-1 (HTLV-1)-infected T-cell lines derived from patients with adult T-cell leukemia (ATL) and HTLV-1-associated myelopathy/tropical spastic paraparesis (HAM/TSP). **a** Expression of CXCL10 mRNA was examined by real-time PCR in HTLV-1-infected and -uninfected T-cell lines. CXCL10 mRNA was preferentially expressed in HTLV-1-infected human T-cell lines derived from patients with ATL (3 out of 5 tested) and HAM/TSP (4 out of 4 tested), compared with HTLV-1-transformed T-cell lines (0 out of 3) and HTLV-1 negative human T-cell lines (0 out of 3). **b** The expressions of *tax* and *HBZ* mRNA were examined by quantitative real-time PCR in HTLV-1-infected T-cell lines. The expression levels of the viral RNAs *tax* and *HBZ* were relatively high in T-cell lines derived from patients with HAM/TSP and HTLV-1-transformed T-cell lines when compared with those in ATL cell lines. **c** CXCL10 levels in culture supernatants from HTLV-1-infected and -uninfected human T-cell lines were assessed by ELISA. Significant levels of CXCL10 was observed in the cultured supernatants from HTLV-1-infected human T-cell lines derived from patients with ATL and HAM/TSP, whereas it was not detectable in any of the other cell lines tested. **d** Expression of Tax and HBZ was examined by Western blot in HTLV-1-infected and -uninfected T-cell lines

We measured the levels of CXCL10 mRNA and protein expression in HTLV-1-infected and -uninfected human T-cell lines. As shown in Fig. 5a, CXCL10 mRNA was preferentially expressed in HTLV-1-infected human T-cell lines derived from patients with ATL (3 out of 5 tested) and HAM/TSP (4 out of 4 tested), compared with HTLV-1-transformed T-cell lines (0 out of 3) and HTLV-1 negative human T-cell lines (0 out of 3). Quantitative real-time PCR and ELISA analysis revealed that, although the expression levels of the viral RNAs *tax* and *HBZ* in ATL-derived cell lines were low (Fig. 5b), high levels of CXCL10 mRNA (Fig. 5a) and protein expression (Fig. 5c) were observed in ATL cell lines, suggesting that over-expression of CXCL10 in ATL-derived cell lines was independent of *tax* gene expression. Western blot also revealed that over-expression of CXCL10 in ATL-derived cell lines was independent of Tax protein expression (Fig. 5d). Importantly, consistent with the cell line data, the expression levels of *CXCL10* mRNA in PBMCs of HAM/TSP patients were also not significantly correlated with the *tax* expression (*p* = 0.4247, *r *= − 0.406 by Spearman rank correlation analysis), indicating that increased *CXCL10* expression is independent of *tax* expression. Thus, our data indicated that increased expression of *CXCL10* mRNA is associated with “having HTLV-1 subgroup-A or subgroup-B”, not “tax expression in infected cells” both in vitro and ex vivo (i.e., in unmanipulated PBMCs obtained from HAM/TSP patients).

### No difference in subgroup-specific Tax molecules with respect to transcriptional activation of the CXCL10 promoter via NF-κB

**Fig. 6:**
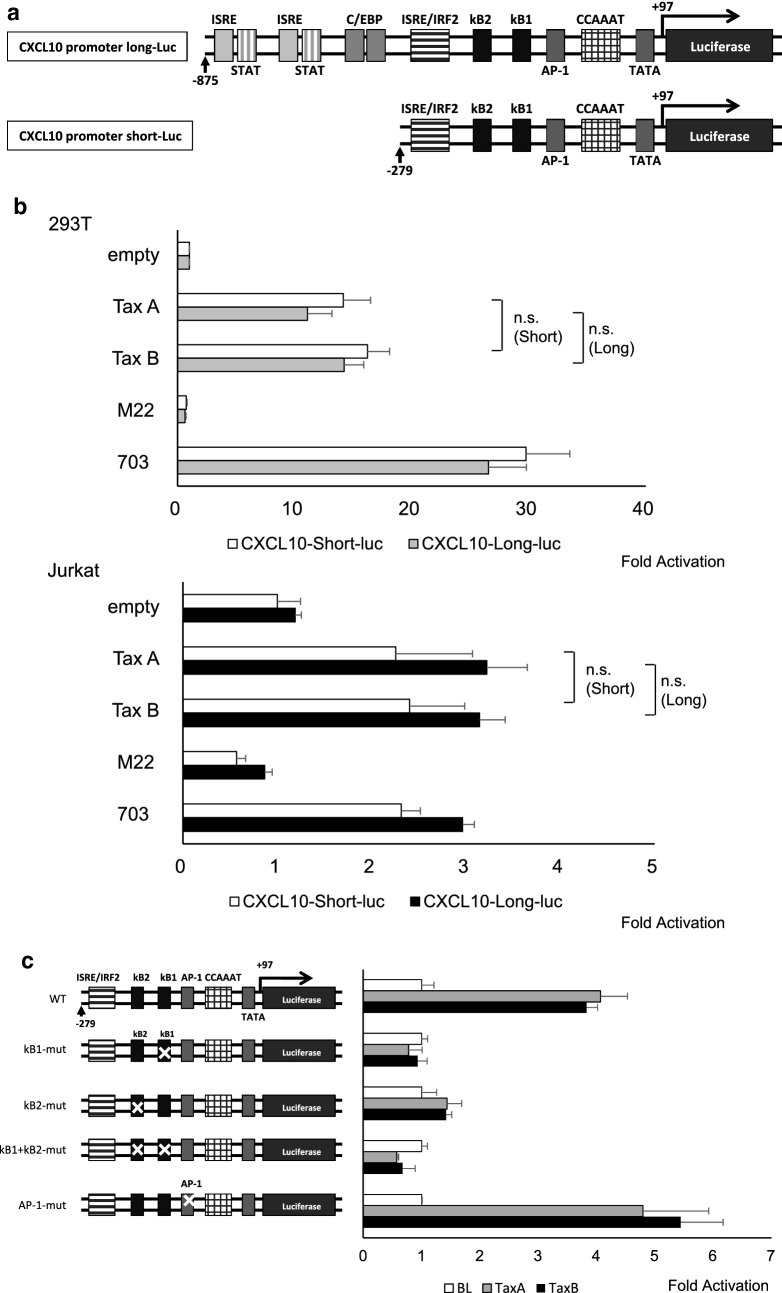
Subgroup-specific Tax molecules do not differ in the transcriptional activation of the CXCL10 promoter via NF-κB. **a** Schematic representation of the CXCL10 promoter sequence cloned into pGL3-Basic. The firefly luciferase gene was used to monitor the activity of the CXCL10 gene promoter. **b** These reporter constructs were independently transfected into Jurkat human T-cells and 293T cells with or without the Tax expression plasmid. Luciferase assays were performed 24 h after transfection. There was no difference between Tax-A and Tax-B with respect to transcriptional activity for both the short and long CXCL10 promoters. The data also showed that the Tax mutant M22, which is defective in NF-κB activation, failed to activate the CXCL10 promoters (both short and long). In contrast, the Tax 703 mutant, which can activate NF-κB but not CREB, efficiently activated the CXCL10 promoters (both short and long). **c** To determine whether either the NF-κB or the AP-1 sequence was required for Tax-mediated activation of the CXCL10 promoter, mutant reporter constructs were co-transfected along with the Tax expression plasmid, and luciferase activity was determined for each of the four mutants. Tax-induced luciferase activity was significantly reduced by mutation in one of the two NF-κB binding site sequence, but not reduced by mutation in the AP-1 site, indicating that Tax transactivation of the CXCL10 involves both κB binding sites. Three independent experiments were performed. Data shown as mean ± SD, n  =  3

We further examined whether there were any differences in the ability of subgroup-specific Tax to activate the human CXCL10 promoter [22]. Jurkat cells were co-transfected with Tax effector plasmids and a reporter gene construct containing the region between − 875 and +97 nucleotides (pGL3-CXCL10-Long-Luc) and − 279 and + 97 nucleotides (pGL3-CXCL10-Short-Luc) of the CXCL10 upstream regulatory sequences (Fig. 6a). After 24 h transfection, Tax-A or Tax-B protein was induced by adding Dox for 24 h. Then, the reporter gene assay was carried out for Tax-mediated transcriptional activation. As shown in Fig. 6b, there was no difference between Tax-A and Tax-B with respect to transcriptional activity for both the short and long CXCL10 promoters in the epithelial cell line 293T and the T cell line, Jurkat. The data also showed that the Tax mutant M22 [34], which is defective in NF-κB activation, failed to activate the CXCL10 promoters (both short and long) (Fig. 6b). In contrast, the Tax 703 mutant [35], which can activate NF-κB but not CREB, efficiently activated the CXCL10 promoters (both short and long) (Fig. 6b). To further determine whether either the NF-κB or the AP-1 sequence was required for Tax-mediated activation of the CXCL10 promoter, we constructed reporter plasmids by site-directed mutagenesis. Then mutant reporter constructs were co-transfected along with the Tax expression plasmid, and luciferase activity was determined for each of the four mutants (Fig. 6c). As a result, Tax-induced luciferase activity was significantly reduced by mutation in one of the two NF-κB binding site sequence, but not reduced by mutation in the AP-1 site, indicating that Tax transactivation of the CXCL10 involves both κB binding sites. Namely, Tax-induced activation of NF-κB pathway is responsible for the upregulation of CXCL10 expression, but subgroup-specific Tax molecules have similar effects on the transcriptional activity of the CXCL10 promoter. This was also the case for another reporter construct containing NF-κB binding site sequence. As shown in Additional file 3: Fig. S1, subgroup-specific Tax molecules do not differ in the transcriptional activation of the reporter construct containing a luciferase gene under control of the NF-κB binding sequence of the IL-2Ra gene [25].

### No difference in Tax-A and Tax-B with respect to binding to NF-κB proteins

**Fig. 7:**
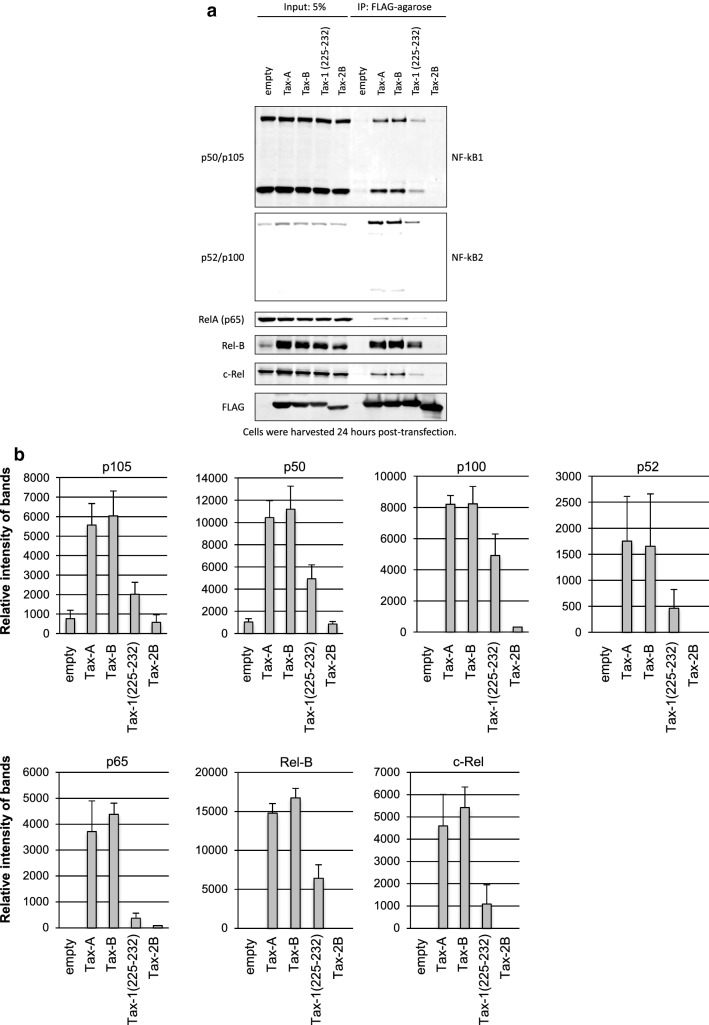
Tax-A and Tax-B do not differ in their binding to NF-κB proteins. To test whether one of the two amino acid differences between Tax-A and Tax-B, i.e., at position 221, which are located just beside the Tax1 (225-232) motif is involved in the p100 processing, we tested whether this amino acid difference affects the binding affinity to the NF-кB protein, thereby altering the ability to activate the NF-кB pathway and their downstream target genes. Jurkat cells were transfected with each expression plasmid and cells were harvested after 24 h, then immunoprecipitation and western blot analyses were performed. **a** Western blot analysis performed on immunoprecipitated Tax protein revealed that each subgroup of Tax, i.e., Tax-A and -B, interacted with each NF-κB components, i.e., c-Rel, RelA, RelB, p50/p105, and p52/p100, with similar efficiencies. **b** The amounts of p100, p52, RelB, p65, p105, p50, and c-Rel proteins in immunoprecipitated samples were quantified by densitometric scanning of corresponding bands of the western blot using Image J software. Vertical bars indicate mean ± SD of the densitometric analysis from four independent experiments

While HTLV-1 is the causative agent of ATL and HAM/TSP, the closely related virus HTLV-2 is not clearly associated with a known clinical disease [36]. It has been reported that HTLV-1 Tax (i.e., Tax1) but not HTLV-2 Tax (Tax2) interacts with NF-кB2/p100/p52 and RelB, and this interaction was mediated by the Tax1 amino acid region 225-232 [37]. As one of the two sites of amino acid differences between Tax-A and Tax-B is located just beside this region (i.e., at position 221), we tested whether this amino acid difference affects binding affinity to NF-кB protein, thereby altering the ability to activate the NF-кB pathway and its downstream target genes. Jurkat cells were transfected with each Tax expression plasmid and cells were harvested after 24 h, then western blot and immunoprecipitation analyses were performed. The amounts of p100, p52, RelB, p65, p105, p50, and c-Rel proteins in immunoprecipitated samples were quantified by densitometric scanning of corresponding bands of the western blot. As shown in Fig. 7a, b, western blot analysis performed on immunoprecipitated Tax protein revealed that each subgroup of Tax (i.e., Tax-A and -B) interacted with each NF-κB component (i.e., c-Rel, RelA, RelB, p50/p105, and p52/p100) with similar efficiency.

### Higher abundance of the DNA fragment bound by ternary complex including Tax-A than that including Tax-B in the CXCL10 promoter

**Fig. 8:**
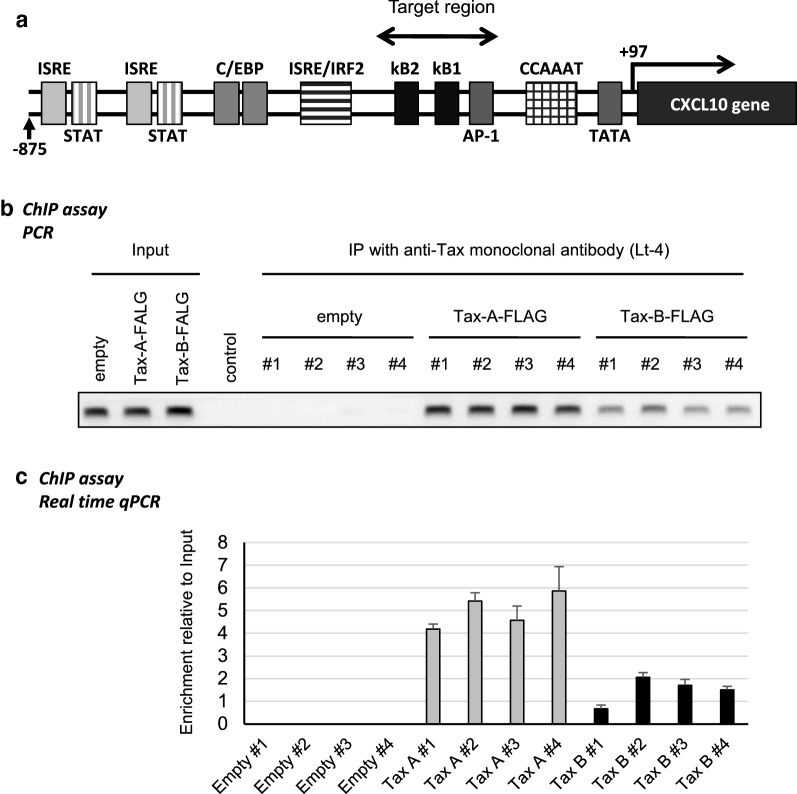
DNA fragment bound by the ternary complex including Tax-A is more abundant than that including Tax-B in the CXCL10 promoter. To investigate whether HTLV-1 subgroups affects the binding of Tax to the endogenous CXCL10 promoter, we performed chromatin immunoprecipitation (ChIP) assays. Chromatin fragments were prepared from Jurkat T-cells transfected with plasmid expressing Tax and immunoprecipitated with specific anti-Tax monoclonal antibody (Lt-4). The immunoprecipitated DNA was then amplified by PCR. **a** Schematic representation of the CXCL10 promoter sequence. “Target region”, which was amplified by PCR, includes two NF-κB sites and an AP-1 site. **b**, **c** Chromatin immunoprecipitation (ChIP) assays. Representative examples of PCR products obtained from chromatin material that was directly immunoprecipitated with anti-Tax monoclonal antibody (Lt-4) (**b**). The ternary protein complex including Tax was found to be associated with the CXCL10 proximal promoter sequence including the two NF-κB sites and an AP-1 site. The abundance of a specific DNA fragment bound by the ternary complex, including Tax, is higher for Tax-A than for Tax-B. Cell lysates were subjected to ChIP assays using Lt-4 antibody followed by qRT-PCR using the specific primer sets (**c**). Three independent experiments were performed. Data shown as mean ± SD

To investigate whether HTLV-1 subgroups affect the binding of Tax to the endogenous CXCL10 promoter, we performed ChIP assays (Fig. 8). Chromatin fragments were prepared from Jurkat T-cells transfected with plasmid expressing Tax and immunoprecipitated with specific anti-Tax monoclonal antibodies (Lt-4). The immunoprecipitated DNA was then amplified via PCR using specific primer pairs that amplify the CXCL10 proximal promoter sequence including the two NF-κB sites and an AP-1 site (Fig. 8a, “Target region”). As shown in Fig. 8a, the ternary protein complex including Tax was found to be associated with the CXCL10 proximal promoter sequence including the two NF-κB sites and an AP-1 site (Fig. 8a). Moreover, the abundance of a specific DNA fragment bound by ternary complex including Tax is higher for Tax-A than for Tax-B (Fig. 8b, c).

## Discussion

In this study, to investigate the role of HTLV-1 subgroups in viral pathogenesis, we first performed a microarray-based study to define the gene expression profiles of each subgroup-specific Tax or HBZ. Our results showed that some Tax-responsive genes identified by microarray, such as EBI3 [26], VCAM-1 [27], IL-13 [28], and CCL1 [29] have already been identified as Tax target genes in previous studies, validating our gene induction system. Furthermore, Tax-induced expression of CXCL10 gene identified by microarray was confirmed by qRT-PCR.

Our microarray analysis showed that out of a total of 50,599 genes including Entrez genes and lincRNA genes screened, approximately 2619 and 8074 were differentially expressed upon Tax or HBZ induction, respectively. The list of the top 50 genes up-regulated by each subgroup of Tax (i.e., Tax-A and -B) or HBZ (i.e., HBZ-A and -B) showed that the genes that were differentially expressed in the presence of Tax included those related to apoptosis (BCL2-related protein A1: BCL2A1), the cell cycle and DNA repair (growth arrest and DNA-damage-inducible, gamma: GADD45G), cytokines (IL3, IL13, IL36G), chemokines (CXCL9, CXCL10), and adhesion molecules (vascular cell adhesion molecule 1: VCAM1). In contrast, the genes that were differentially expressed in the presence of HBZ include those related to RNA-splicing (RNA, U6atac small nuclear: RNU6ATAC), the Notch signaling pathway (NOTCH-regulated ankyrin repeat protein: NRARP) and chemokine (CCL21). These results suggest that Tax mainly induces expression of genes related to the activation and transformation of CD4+ T cells, whereas HBZ modulates a variety of cellular signaling pathways which are related to the immune response, differentiation, and growth of T-cells.

Our microarray results also showed that various kinds of ncRNAs frequently appeared among both Tax- and HBZ-regulated genes. It is now well established that the majority of transcribed genomic sequences are associated with ncRNAs rather than protein-coding RNAs, and such ncRNAs, including regulatory miRNAs and lncRNAs that are functionally involved in a variety of host immune responses and in the pathogenesis of human diseases [38]. It is, therefore, plausible to propose that deregulation of the ncRNA signature caused by a virus infection will strongly affect the phenotype and function of the infected cell. Indeed, in HTLV-1 infection, it has been reported that the expression of miR-31, which is a negative regulator of the noncanonical NF-κB pathway, was genetically and epigenetically silenced in ATL cells, resulting in constitutive NF-κB activation [39]. In addition, lncRNAs, a heterogeneous class of RNAs defined as non-protein-coding transcripts longer than 200 nucleotides, are thought to play a role in proteasomal and ubiquitination pathways, apoptosis, DNA damage responses, and cell cycle regulation [40]. lncRNAs have been directly linked to human diseases such as certain cancers and autoimmune and neurodegenerative diseases [40]. However, to date, the role of lncRNAs in viral infections remains largely unknown. Our results provide new insights into the hitherto unknown functions of lncRNAs in infection by viruses including HTLV-1.

A previous report suggested that serum CXCL10 is significantly higher in HAM/TSP patients than in HCs, and the CSF level of CXCL10 was strongly correlated with disease severity [32]. Thus, CXCL10 concentration was proposed as a potential prognostic biomarker for HAM/TSP. Our data showed that among the Tax-regulated target genes, CXCL10 was approximately 1.5 times more strongly induced by Tax-A than Tax-B. More importantly, real-time qRT-PCR on PBMCs obtained from HAM/TSP patients indicated that the expression levels of CXCL10 in HAM/TSP patients with subgroup-A were significantly higher than those in HAM/TSP patients with subgroup-B. The difference in transcription is likely to be due to a difference in the action of the NF-κB/Rel family of transcription factors. In HTLV-1 infection, Tax-mediated NF-κB activation is recognized as a crucial factor associated with the development of HTLV-1-associated diseases [41], since NF-κB, which consists of five molecules (RelA (p65), RelB, c-Rel, p50, and p52) that form transcriptionally active complexes in various combinations, has an essential role in inflammation, innate immunity, and many steps of cancer initiation and progression [42]. Indeed, ATL cells and their derivative cell lines carry constitutively active NF-κB regardless of their Tax expression, and NF-κB is required for immortalization and also the survival of HTLV-1 transformed cells [43]. We therefore determined whether the HTLV-1 viral protein Tax activates the expression of CXCL10 at the transcriptional level and whether there were any differences in the ability of subgroup-specific Tax molecules to activate the CXCL10 promoter. However, contrary to our expectations, there was no difference in the ability of each subgroup Tax to activate the CXCL10 promoter, although transient Tax expression in an HTLV-1-negative human T-cell line activated the CXCL10 gene promoter through the NF-κB pathway.

In clear contrast to HTLV-1, HTLV-2 has not been associated with ATL or other types of malignancies [36]. As HTLV-1 Tax (Tax1) and HTLV-2 Tax (Tax2) have many shared activities but also certain significantly distinct activities [36], the difference between those two Tax proteins may reveal the key roles in HTLV-1-induced malignant transformation. Most importantly, although Tax2 activates the classical pathway of NF-κB, similar to Tax1, malignant transformation by Tax2 has rarely been reported [44]. Thus, one significant difference between Tax1 and Tax2 is the activation of transcription factor NF-κB2/p100/p52, which is a key player in the alternative, non-classical NF-κB pathway [44]. Interestingly, Tax1 but not Tax2 was reported to interact with NF-κB2/p100/p52 and RelB, and the distinct interaction activity was mediated by the Tax1 amino acid region 225-232, and one of the two sites of amino acid differences between Tax-A and Tax-B is located just beside this region (i.e., at position 221). We therefore tested whether this amino acid difference affected the binding affinity to NF-кB protein, thereby altering the ability to activate the NF-кB pathway and their downstream target genes. The results showed that both Tax-A and Tax-B showed similar levels of respective binding to each of the NF-κB proteins, i.e., NF-κB1 (p105/p50), NF-κB2 (p100/p52), RelA (p65), RelB, and c-Rel. This is consistent with the CXCL10 reporter gene assay, which revealed no difference in the ability of each subgroup Tax to activate the CXCL10 promoter via NF-κB pathway as a transcriptional regulator.

To further investigate how the subgroup-specific Tax regulates the CXCL10 gene expression in T-cells, we performed the ChIP assay using Jurkat T-cells. The results showed that DNA fragments bound by ternary protein complexes including Tax-A are more abundant than those including Tax-B in the CXCL10 promoter, suggesting that Tax-A is more frequently recruited at this genomic region than Tax-B.

In conclusion, we demonstrate that different HTLV-1 subgroups are characterized by different patterns of host gene expression. Differential expression of pathogenesis-related genes regulated directly or indirectly by subgroup-specific Tax or HBZ may be associated with the onset of HAM/TSP. To better understand the pathogenesis of HAM/TSP, further studies are needed to elucidate the role of subgroup-specific viral transcription factors and its target genes in more detail.

## Additional files

**Additional file 1: Table S1.** Osame Motor Disability Score (OMDS).

**Additional file 2: Table S2.** Primer sequences for plasmid construction.

**Additional file 3: Fig. S1.** Subgroup-specific Tax molecules do not differ in the transcriptional activation of the reporter construct containing a luciferase gene under control of the NF-κB binding sequence of the IL-2Ra gene. The reporter construct containing a luciferase gene fused to five repeats of the NF-κB site of the IL-2Ra gene was independently transfected into Jurkat human T-cells with or without the Tax expression plasmid. Luciferase assays were performed 24 h after transfection. There was no difference between Tax-A and Tax-B with respect to transcriptional activity. Three independent experiments were performed. Data shown as mean ± SD, n  =  3.

## Abbreviations

HTLV-1: human T-cell leukemia virus type-1
ATL: adult T-cell leukemia
HAM/TSP: HTLV-1-associated myelopathy/tropical spastic paraparesis
HCs: asymptomatic carriers
NCs: normal uninfected healthy controls
CSF: cerebrospinal fluid
PVL: proviral load
CNS: central nervous system
