# Supplementary material for: Distinct gene expression signatures induced by viral transactivators of different HTLV-1 subgroups that confer a different risk of HAM/TSP
Source: Retrovirology. 2018 Nov 6;15:72. doi: 10.1186/s12977-018-0454-x (PMC6219256; doi:10.1186/s12977-018-0454-x)
Supplement: Supplementary file 1 — Additional file 1: Table S1. Osame Motor Disability Score (OMDS). [file 12977_2018_454_MOESM1_ESM.docx]

## Additional file 1: Table S1. Osame Motor Disability Score (OMDS).

| Grade | Motor disability |
| --- | --- |
| 0 | Normal gait and running |
| 1 | Normal gait but runs slowly |
| 2 | Abnormal gait (staggering or spastic) |
| 3 | Abnormal gait and unable to run |
| 4 | Needs support while using stairs but walks without assistance |
| 5 | Needs one hand support in walking |
| 6 | Needs two hands support in walking (can walk more than 10 meter) |
| 7 | Needs two hands support in walking (can walk less than 10 meter) |
| 8 | Needs two hands support in walking (can walk less than 5 meter) |
| 9 | Unable to walk but can walk on all fours |
| 10 | Unable to walk on all fours but can crawl with hands |
| 11 | Unable to crawl with hands but can turn sideways in bed |
| 12 | Unable to turn sideways but can move the toes |
| 13 | Completely bedridden (unable to move the toes) |
