# Supplementary material for: Distinct gene expression signatures induced by viral transactivators of different HTLV-1 subgroups that confer a different risk of HAM/TSP
Source: Retrovirology. 2018 Nov 6;15:72. doi: 10.1186/s12977-018-0454-x (PMC6219256; doi:10.1186/s12977-018-0454-x)
Supplement: Supplementary file 2 — Additional file 2: Table S2. Primer sequences for plasmid construction. [file 12977_2018_454_MOESM2_ESM.docx]

## Additional file 2: Table S2: Primer sequences for plasmid construction.

| Primer name | Direction | Sequences (5' to 3') |
| --- | --- | --- |
| Tax-FOR | Forward | CGCACGC**GTCGAC**GCCACCACCATGGCCCACTTCCCAGGGTTTG |
| Tax-REV | Reverse | TGGA**AGATCT**CAGACTTCTGTTTCGCGGAAATG |
| HBZ-FOR | Forward | ACGCACGC**GTCGAC**GCCACCACCATGGCGGCCTCAGGGCTGT |
| HBZ-REV | Reverse | TGGA**AGATCT**TATTGCAACCACATCGCCTC |
| Tax-Stop-REV | Reverse | TGGA**AGATCT**CAGACTTCTGTTTCGCGGAAATG |
| Tax-FLAG-REV | Reverse | TCGTCGTCATCCTTGTAGTCTTTACCGACTTCTGTTTCGCGGAAATG |
| FLAG-Tandem-REV | Reverse | GTCATCCTTGTAGTCCCCCTTGTCGTCGTCATCCTTGTAGTCTT |
| FLAG-Tandem-NotI-REV | Reverse | TGCATGC**GCGGCCGC**CTACTTGTCGTCGTCATCCTTGTAGTCCC |
| Tax-2-FOR | Forward | CGCACGC**GTCGAC**GCCACCACCATGGCCCATTTCCCAGGATTCG |
| Tax-2-FLAG-Tandem-REV | Reverse | TCGTCGTCATCCTTGTAGTCTTTACCCTTGGGATTGTTTGTGTGAGA |
| CXCL10-875-FOR | Forward | GCGTA**GGTACC**GAACCCCATCGTAAATCAACCTG |
| CXCL10-279-FOR | Forward | GCGTA**GGTACC**AGAATGGATTGCAACCTTTG |
| CXCL10-REV | Reverse | CGCACGC**CTCGAG**GCAGCAAATCAGAATGGCAGTTTG |
| kB1-mut FOR | Forward | TGCAACATG**T**GACTTC**A**CCAGGAACA |
| kB1-mut REV | Reverse | TGTTCCTGG**T**GAAGTC**A**CATGTTGCA |
| kB2-mut FOR | Forward | GGAGCAGAG**T**GAAATT**A**CGTAACTTG |
| kB2-mut REV | Reverse | CAAGTTACG**T**AATTTC**A**CTCTGCTCC |
| AP1-mut FOR | Forward | GGTTTTGCTAAG**AG**AACTGTAATGCC |
| AP1-mut REV | Reverse | GGCATTACAGTT**CT**CTTAGCAAAACC |

GTCGAC, AGATCT, GCGGCCGC, GGTACC, and CTCGAG sequences are the respective restriction sites for SalI, BglII, NotI, KpnI, and XhoI.

Underlines indicate the FLAG-tag sequences. Double underlines indicate the Kozak sequences.

For “κB1-mut”, “κB2-mut” and “AP-1 mut”, bold letters indicate mutated sequence.
