# Supplementary figures and images for: Distinct gene expression signatures induced by viral transactivators of different HTLV-1 subgroups that confer a different risk of HAM/TSP
Source: Retrovirology. 2018 Nov 6;15:72. doi: 10.1186/s12977-018-0454-x (PMC6219256; doi:10.1186/s12977-018-0454-x)

## Slide 1
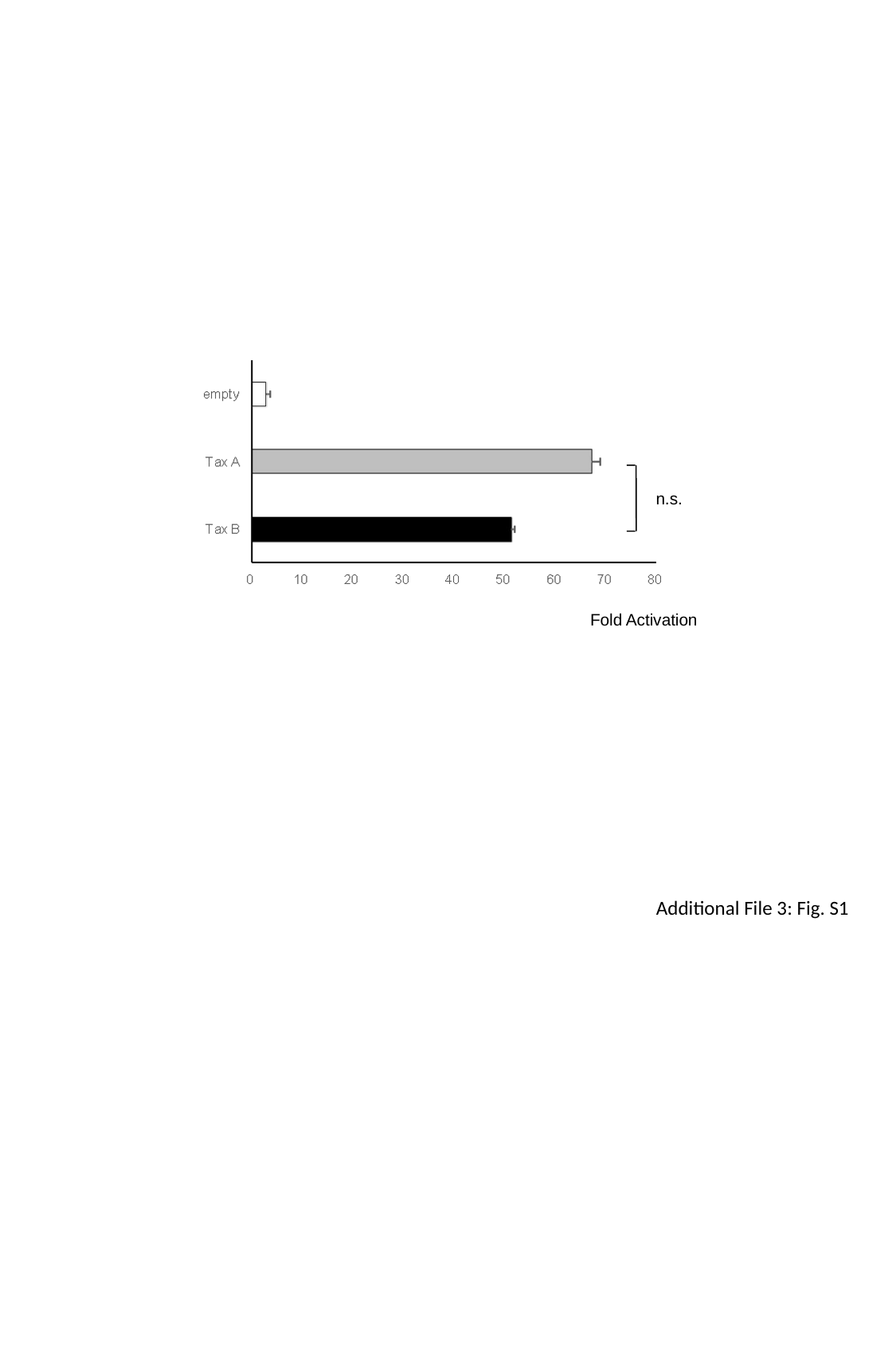

n.s.
Fold Activation
Additional File 3: Fig. S1

Supplement: Supplementary file 3 — Additional file 3: Fig. S1. Subgroup-specific Tax molecules do not differ in the transcriptional activation of the reporter construct containing a luciferase gene under control of the NF-κB binding sequence of the IL-2Ra gene. The reporter construct containing a luciferase gene fused to five repeats of the NF-κB site of the IL-2Ra gene was independently transfected into Jurkat human T-cells with or without the Tax expression plasmid. Luciferase assays were performed 24 h after transfection. There was no difference between Tax-A and Tax-B with respect to transcriptional activity. Three independent experiments were performed. Data shown as mean ± SD, n = 3. [file 12977_2018_454_MOESM3_ESM.ppt]
